# Differential Effects of Sleep Respiratory Event Types on Heart Rate Variability: Central Apnea as the Most Significant

**DOI:** 10.3390/diagnostics16121770

**Published:** 2026-06-08

**Authors:** Tianci Zhao, Cong Fu, Wei Chen, Chen Chen, Huan Yu

**Affiliations:** 1 Department of Biomedical Engineering, College of Biomedical Engineering, Fudan University, Shanghai 200433, China; 23210720084@m.fudan.edu.cn; 2 Department of Neurology, Huashan Hospital, Fudan University, Shanghai 200040, China; applepie_cong@126.com; 3 Sleep and Wake Disorders’ Center of Fudan University, Shanghai 200040, China; 4 National Center for Neurological Disorders, Shanghai 200040, China; 5 School of Biomedical Engineering, The University of Sydney, Camden, NSW 2570, Australia; wei.chenbme@sydney.edu.au; 6 Center for Medical Research and Innovation, Shanghai Pudong Hospital, Shanghai 201203, China; 7 Human Phenome Institute, Fudan University, Shanghai 201203, China

**Keywords:** central apnea, ultra-short-term HRV, autonomic function, sleep-disordered breathing

## Abstract

**Background:** Sleep-disordered breathing (SDB) is frequently accompanied by autonomic nervous system (ANS) dysfunction, which is closely associated with an increased incidence of cardiovascular diseases and elevated mortality risk. Heart rate variability (HRV) serves as a classic metric for evaluating sympathovagal balance; however, the specific impacts of four distinct types of respiratory events—obstructive apnea (OA), central apnea (CA), mixed apnea (MA), and hypopnea (HYP)—on HRV remain underinvestigated. Utilizing ultra-short-term HRV analysis, this study aimed to evaluate the immediate effects of different respiratory events on ANS function, while further exploring the modulatory roles of arousal, Apnea–Hypopnea Index (AHI) severity and sleep stages (non-rapid eye movement [NREM] vs. rapid eye movement [REM]). **Methods:** A total of 108 patients with SDB undergoing overnight polysomnography (PSG) were included. A total of 19,862 respiratory events, including obstructive apnea (OA), central apnea (CA), mixed apnea (MA), and hypopnea (HYP), were analyzed using 15 s ECG segments. Linear mixed-effects models (LMMs) and estimated marginal means (EMMs) with Sidak-adjusted pairwise comparisons were constructed to evaluate differences in ECG-derived features and to analyze differences between event types. **Results:** Central apnea (CA) was associated with significantly reduced HRV and heart rate indices, including Standard Deviation of Successive Differences (SDSD), Root Mean Square of the Successive (RMSSD), Standard Deviation 1 (SD1), and heart rate (HR), compared with other respiratory event types (all *p* < 0.05). Across all event types, HRV metrics exhibited consistent dynamic changes before, during, and after respiratory events (all *p* < 0.001), characterized by a decrease during the event followed by post-event recovery. In the interaction effect of sleep stage, SDSD was significantly lower in CA compared with both OA (estimate = −11.67, 95% CI −18.78 to −4.59, *p* < 0.001) and HYP (estimate = −11.38, 95% CI −18.55 to −4.20, *p* < 0.001) during NREM sleep. No significant differences in HRV parameters, heart rate, or QRS duration were observed between OA and HYP (all *p* > 0.05). **Conclusions:** This study is the first to elucidate the differential impacts of four distinct types of sleep respiratory events on ultra-short-term HRV, confirming that CA events exert the most profound effects on autonomic function. These findings suggest that the proportion of CA occurrences could serve as a more precise biomarker for identifying individuals at high risk for cardiovascular diseases within the SDB population.

## 1. Introduction

With the overall prevalence rate of 20% in the community population, SDB is a highly prevalent sleep-related disease globally [1]. Some studies estimate that the number of adults aged 30–69 years old suffered from moderate-to-severe obstructive sleep apnea (OSA) alone could be as high as 425 million worldwide [2]. It has been confirmed that the large population of SDB patients increases the risks of heart failure [3], cardiac arrhythmias (such as nocturnal atrial fibrillation, sinus arrest, atrioventricular block, etc.) [4], myocardial infarction, and death [5]. Part of this causal link is mediated by the autonomic nervous system (ANS) [6,7]. Patients with SDB exhibit alterations in sympatho-vagal balance during sleep [8,9], and these alterations persist during the daytime [10,11].

Although the Apnea–Hypopnea Index (AHI) is routinely used to quantify the severity of sleep-disordered breathing (SDB), it provides only a summary measure of event frequency and does not differentiate among the distinct types of respiratory disturbances defined by the AASM manual. Obstructive apnea, central apnea, mixed apnea, and hypopnea arise from different pathophysiological mechanisms and elicit different physiological stresses, including variations in respiratory effort, intrathoracic pressure swings, chemoreflex activation, and arousal propensity. These differences suggest that each event subtype may induce a unique pattern of autonomic nervous system (ANS) activation. Therefore, reliance on AHI alone may obscure important heterogeneity in autonomic responses, limiting our understanding of how specific respiratory event types contribute to ANS dysfunction and SDB-related cardiovascular risk.

HRV is a non-invasive tool for assessing autonomic activity, encompassing various electrophysiological signal processing and analysis methods such as time-domain, frequency-domain, and non-linear analyses [12]. It has significant value in evaluating the autonomic functional status of SDB patients [13,14,15], assisting in disease diagnosis [16,17], and predicting cardiovascular disease risk [18]. Generally, based on the length of the time window, HRV analysis can be divided into short-term (5–15 min) or long-term (1–24 h). Long-term analysis is typically used to assess patient mortality and adverse health outcomes, while short-term analysis is suitable for tracking dynamic changes [12]. Ultra-short HRV (usHRV) involves analyzing HRV changes within an ultra-short time window (<5 min electrocardiogram (ECG) samples) [19] and can be used to predict the risk of sudden cardiac death [20]. In the field of SDB, usHRV is the only method for analyzing changes in ANS associated with a single respiratory event [21,22]. SDNN and RMSSD are commonly used statistical metrics; the former reflects all cyclic components contributing to heart rate variability and primarily represents overall autonomic variability, while the latter reflects short-term rapid fluctuations in heart rate, representing the regulatory function of the parasympathetic nervous system (vagus nerve) on the heart and the balance status of the ANS [23].

However, despite increasing evidence linking SDB to autonomic dysfunction, several important limitations remain in the current literature. First, many previous studies have primarily relied on the AHI as the principal indicator of disease severity, without considering the potential heterogeneity of autonomic responses induced by different respiratory event subtypes. As obstructive apnea, central apnea, mixed apnea, and hypopnea involve distinct physiological mechanisms, combining them into a single frequency-based metric may obscure subtype-specific ANS alterations. Second, conventional HRV analyses are commonly performed using relatively long time windows, which are insufficient for capturing the rapid and transient autonomic fluctuations associated with individual respiratory events during sleep. Although ultra-short HRV analysis provides a promising approach for evaluating event-level autonomic responses, existing studies remain limited, and the differential autonomic effects among respiratory event subtypes have not been fully characterized. Therefore, further investigation focusing on subtype-specific respiratory events and their associated ultra-short-term HRV changes may improve the understanding of ANS dysregulation in SDB and provide additional insights into cardiovascular risk stratification.

This study utilizes an ultra-short time window to capture the dynamic changes in HRV before and after a single respiratory event. To the best of our knowledge, it is the first time the differences in the immediate impact of different respiratory event subtypes on ANS have been explored, and thus the first time the modulating roles of AHI severity and sleep stages have been observed.

## 2. Materials and Methods

### 2.1. Participants

The study included a total of 113 patients who were diagnosed with SDB for the first time at the Sleep Center of Huashan Hospital between January 2020 and June 2024. Among them were 99 males and 9 females. All experienced OA events and CA events during sleep. Each participant provided informed consent, and this combined retrospective and prospective cohort study was approved by the Human Ethics Committee of Huashan Hospital, Fudan University (HIRB) [Ethics Approval Number: 2021-811-X1].

Demographic data and vital signs of the subjects were assessed during the study. Clinical data for each subject were collected via standardized semi-structured questionnaires, including age, gender, body mass index (BMI), medical history, current medications, sleep disorders, Epworth Sleepiness Scale, FS-14 Fatigue Scale, Beck Anxiety Inventory, and Beck Depression Inventory. All subjects completed a standard Type I overnight polysomnography (PSG). HRV analysis was performed on the data from 113 cases; 5 patients were excluded due to ECG artifacts exceeding 20% of the complete data, resulting in a final set of ECG samples from 108 subjects.

### 2.2. Video-Synchronized PSG

All subjects completed one night of PSG monitoring (Compumedics Grael series, Australia) at the Sleep Center. The recorded leads included F3/F4, C3/C4, and O1/O2 electroencephalogram (EEG); bilateral electrooculogram (EOG); submental/leg electromyogram (EMG); ECG; oronasal airflow; thermal airflow; thoracic and abdominal respiratory effort; and pulse oximetry. PSG analysis was performed manually by professional sleep technicians. All event scoring criteria followed the 2020 American Academy of Sleep Medicine (AASM) Manual for the Scoring of Sleep and Associated Events, Version 2.6 [24]. An apnea event was defined as a drop in the peak signal excursion by ≥90% of the pre-event baseline for a duration of ≥10 s. Based on the presence or absence of inspiratory effort, events were classified as OA, CA, or MA. A HYP event was defined as a drop in the peak signal excursion by ≥30% of the baseline for a duration of ≥10 s, accompanied by a ≥3% oxygen desaturation or an arousal at the end of the event.

### 2.3. HRV Analysis

The placement of ECG electrodes followed the AASM Scoring Manual Version 2.6, with a sampling rate of 512 Hz. None of the subjects consumed alcohol or caffeinated beverages on the night of the PSG monitoring. HRV analysis was performed using the single-lead ECG signal collected by the PSG.

This study aims to describe the dynamic changes in the ANS associated with different respiratory phases during sleep. To this end, we performed HRV analysis on ECG signals recorded throughout the sleep period. First, quality control was performed on the raw ECG signals to identify and exclude segments contaminated by significant noise, motion artifacts, or ectopic beats, thereby ensuring the reliability of subsequent analyses. To ensure data quality, we conducted visual inspections for all extracted ECG segments and removed those with obvious artifacts or abnormal rhythms. The proportion of excluded segments was approximately 1% of the total, having a negligible impact on the overall analysis results.

After preprocessing, we employed the classic Pan–Tompkins algorithm to detect and identify R-wave peaks in the filtered ECG waveforms. Subsequently, the occurrence times of these consecutive R-wave peaks were used to construct the R-R interval (RRI) time series, which served as the basis for all subsequent feature extraction.

Since the duration of OA and MA is typically 10–60 s, whereas the duration of CA is 10–30 s, the use of different window lengths would affect the comparison of features. Considering the duration of sleep respiratory events, we used a 15 s analysis window. Previous studies on ultra-short-term HRV have rigorously validated that time-domain metrics reflecting beat-to-beat variance—specifically RMSSD and SDSD—as well as the non-linear metric SD1, can be reliably computed and retain physiological validity in extremely short windows of 10 to 30 s [25,26].

Although the clinical diagnostic criterion for a sleep respiratory event is a minimum duration of 10 s, the actual durations in our dataset predominantly ranged from 20 to 40 s. We explicitly selected a 15 s analysis window (rather than 10 s) as a necessary methodological trade-off: a 10 s window yields an insufficient number of RR intervals (<10 beats) for statistically robust variance calculations, whereas a 15 s window strictly ensures the mathematical reliability of ultra-short-term HRV metrics while remaining fully encompassed within the actual duration of the vast majority of events. Because these specific metrics capture instantaneous, vagally mediated heart rate changes rather than long-term fluctuations, a 15 s window is mathematically adequate. Based on the time markers of respiratory events, 15 s window signals located 7.5 s before and after the midpoint of the respiratory event were extracted from the raw ECG signals to represent the signals during the occurrence of the respiratory events.

Feature extraction from short-term ECG segments: To extract the heart rate variability (HRV) features, the raw electrocardiogram (ECG) data were first exported from the polysomnography (PSG) system. The PSG records were meticulously reviewed and scored by experienced sleep medicine physicians according to standard clinical guidelines. This expert annotation provided precise time markers, including the specific type of sleep-disordered breathing event (e.g., OA, CA, MA) and its exact onset and termination timestamps.

To accurately detect the R-wave peaks in the exported ECG waveforms, we implemented the classic Pan–Tompkins algorithm [27]. Specifically, the raw ECG signals were pre-processed using band-pass filtering (0.5–48 Hz) and differentiation to reduce baseline wander and high-frequency noise, followed by squaring and moving-window integration to enhance the QRS complexes for accurate R-peak detection. Ectopic beats and artifacts were subsequently identified and removed to ensure that only Normal-to-Normal (NN) intervals were used for HRV analysis. Finally, utilizing the synchronized timestamps provided by the clinical annotations, a 15 s ECG signal window—strictly centered around the midpoint of each specific respiratory event—was segmented and extracted to calculate the event-specific HRV metrics.

These features were primarily categorized into three groups to provide a multifaceted assessment of cardiac autonomic control and electrophysiological properties:

Linear HRV features: This category includes time-domain and frequency-domain indices. Time-domain features quantify the magnitude of variability in the RRI series. Key metrics include the Root Mean Square of Successive Differences between NN intervals (RMSSD), which reflects overall and short-term beat-to-beat variability, respectively.

ECG morphological features: In addition to HRV metrics, features were extracted directly from the ECG waveform itself, the QRS duration to reflect the duration of ventricular depolarization and serve as an indicator of cardiac electrical conduction (see Table 1).

### 2.4. Statistical Analysis Methods

All statistical analyses were performed using MATLAB (R2023a) software. All discrete variables were expressed as frequencies and percentages. A total of 108 subjects were included in this study and stratified into three groups according to the apnea–hypopnea index (AHI): Mild (5 ≤ AHI < 15, n = 41), Moderate (15 ≤ AHI < 30, n = 17), and Severe (AHI ≥ 30, n = 50), as summarized in Table 2.

Prior to statistical analysis, all continuous variables were tested for normality using the Shapiro–Wilk test. Variables that satisfied the normality assumption were expressed as mean ± standard deviation (Mean ± SD), while non-normally distributed variables were presented as median and interquartile range (Median, IQR).

#### 2.4.1. Linear Mixed-Effects Modeling

To investigate the differences in physiological characteristics across various types of sleep respiratory events, linear mixed-effects models (LMMs) were constructed. Given that multiple respiratory events could be recorded from a single subject, patient ID was incorporated as a random intercept to account for intra-subject correlation.

In the primary model, physiological features were set as the dependent variables, with respiratory event types (OA, CA, MA, and HYP) included as fixed effects. To adjust for potential confounders, demographic and clinical covariates—specifically age, body mass index (BMI), hypertension, and coronary artery disease (CAD)—were integrated into the model. Furthermore, since cortical arousal (presence vs. absence) is known to influence autonomic regulation, it was also included as a covariate. The primary model was formulated as follows:

(1) Feature ∼ Event Type+Arousal+Age+BMI+Hypertension+CAD+(1∣Patient ID)

To further examine whether the impact of respiratory event types varies under different physiological or clinical conditions, interaction models were constructed.

Specifically, to evaluate whether sleep stages modulate the differences among respiratory event types, sleep stage (REM vs. NREM sleep) and its interaction term with event type were introduced into the model:

(2) Feature∼Event Type∗Sleep Stage+Arousal+Age+BMI+Hypertension+CAD+(1∣PatientID)

To investigate the potential influence of disease severity, patients were stratified into Mild (5 ≤ AHI < 15), Moderate (15 ≤ AHI < 30), and Severe (AHI ≥ 30) groups based on their Apnea–Hypopnea Index (AHI). The interaction between event type and disease severity was examined using the following formula:

(3) Feature∼Event Type∗Severity+Arousal+Age+BMI+Hypertension+CAD+(1∣PatientID)

Stratified and post hoc analyses: All post hoc analyses were performed based on the fitted LMMs. Estimated marginal means (EMMs) were computed from each model to quantify adjusted differences between respiratory event types while accounting for covariates and random effects.

Pairwise comparisons between event types were conducted using EMMs with Sidak-adjusted *p*-values [30]. Additionally, stratified analyses based on sleep stage and disease severity were performed, and EMM-based pairwise comparisons were applied within each subgroup to further characterize condition-specific differences.

#### 2.4.2. Time-Series Feature Analysis

To assess dynamic changes in ECG-derived features before and after respiratory events, time-series segments were extracted from three time windows: 15 s before the onset of each event, a peri-event window centered at the midpoint of the event (7.5 s before and after), and 15 s after the event. Events separated by less than 15 s were excluded to avoid overlap.

Feature values were computed for each time window, and differences across time points were evaluated using the Friedman test for repeated measures. Post hoc pairwise comparisons were conducted with Sidak correction to adjust for multiple testing.

## 3. Results

### 3.1. Characteristics of Participants

This study enrolled 108 subjects, divided into three groups based on the AHI index: Mild group (5 ≤ AHI < 15, n = 41), Moderate group (15 ≤ AHI < 30, n = 17), and Severe group (AHI ≥ 30, n = 50), as shown in Table 2. There was a significant difference in age among the three groups (*p* = 0.002), with the Severe group being the oldest (51.9 ± 13.6 years) and the Mild group the youngest (41.2 ± 13.5 years). No statistically significant differences among the three groups regarding the incidence of comorbidities such as hypertension and coronary heart disease, or the use of related medications (antihypertensives, beta-blockers, benzodiazepines) (*p* > 0.05). However, the incidence of hypertension (38.0%) and the usage rate of beta-blockers (8.0%) in the Severe group were higher than in the Mild group (22.0%, 0%).

No significant differences in gender, BMI, subjective sleepiness, fatigue, and emotional state dimensions among patients with different AHI severities. Compared to the Mild and Moderate groups, the Severe group showed significantly reduced Total Sleep Time (TST) and significantly prolonged Wake After Sleep Onset (WASO). Regarding sleep architecture, the Severe group exhibited increased N1 sleep and decreased N3 and REM sleep. In terms of sleep-related respiratory events, the Severe group had the lowest mean oxygen saturation and the lowest minimum oxygen saturation during sleep, and the longest CT90 duration. Specific parameters are shown in Table 2.

### 3.2. HRV Characteristics of Different Respiratory Event Types

A total of 19,862 respiratory events were selected for HRV analysis, covering four types of respiratory events: OA, 7135 events; CA, 2104 events; MA, 4576 events; and HYP, 6047 events. The results showed significant differences in HRV features among the various respiratory events (*p* < 0.001), as detailed below.

#### 3.2.1. General Comparison of HRV Features

Based on the fitted linear mixed-effects models, estimated marginal means (EMMs) were computed to evaluate differences across respiratory event types. LMM analysis revealed that, after adjusting for covariates, significant differences existed in HRV characteristics across the different types of respiratory events (Table S1). Compared to obstructive apnea (OA), central apnea (CA) events exhibited significant reductions in SDSD, RMSSD, SD1, and HR metrics (all *p* < 0.05).

This suggests that CA exerts a stronger inhibitory effect on the autonomic nervous system and cardiac rhythm, manifesting as a more pronounced decline in HRV and heart rate deceleration. Similarly, mixed apnea (MA) events were associated with decreased SDSD, RMSSD, and SD1 values, while also demonstrating a prolongation of the QRS duration, indicating a more prominent adverse impact on ventricular conduction. In contrast, hypopnea (HYP) events did not exhibit significant differences from OA regarding their effects on autonomic regulation and cardiac electrical conduction. Among the adjusted covariates, CAD demonstrated a significant impact, leading to significantly elevated HRV metrics. Conversely, other factors, including cortical arousal state, exerted no significant independent effects on HRV or HR (all *p* > 0.05).

**Figure 1:**
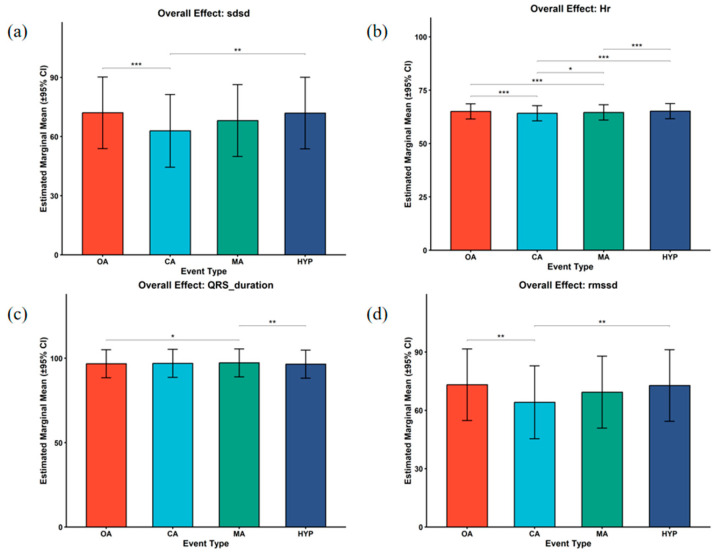
Estimated marginal means (EMMs) of (**a**) SDSD, (**b**) HR, (**c**) QRS duration and (**d**) rmssd across respiratory event types derived from linear mixed-effects models. Error bars represent 95% confidence intervals. Pairwise comparisons were adjusted using the Sidak method. Statistical significance is indicated as * *p* < 0.05, ** *p* < 0.01, *** *p* < 0.001.

Sidak-adjusted pairwise comparisons (Table 3 and Figure 1) further demonstrated that, compared to OA and HYP, CA events were associated with significantly lower RMSSD, SDSD, SD1, and HR. This confirms that CA induces the most profound decline in HRV and heart rate deceleration. Additionally, compared to OA and HYP, the MA group exhibited a significantly prolonged QRS duration, emerging as the only event type to significantly prolong this metric.

#### 3.2.2. Dynamic Changes in HRV Before and After Respiratory Events

**Figure 2:**
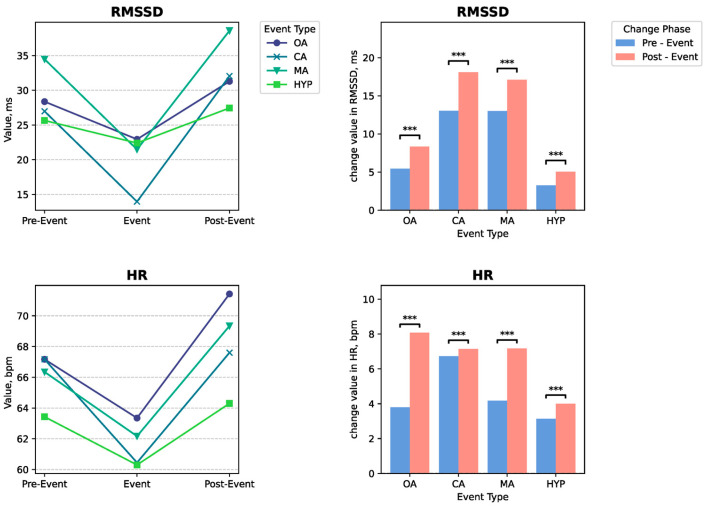
Dynamic changes in cardiac electrophysiology during and surrounding the occurrence of different respiratory event. Figure Legend: Significance markers: *** *p* < 0.001.

HRV metrics across all types of respiratory events exhibited significant dynamic changes across the pre-event, intra-event, and post-event phases (*p* < 0.001, Table 4), displaying highly consistent trend patterns (Figure 2-Left). During the occurrence of respiratory events, the SDSD, RMSSD, and SD1 indices declined, indicating an inhibition of parasympathetic (vagal) tone. Following event termination, these indices rebounded and surpassed their pre-event baselines, suggesting a gradual recovery and even a compensatory surge in parasympathetic function.

Regarding the amplitude of fluctuation (Figure 2—right), using RMSSD as a representative metric, CA and MA events demonstrated the widest fluctuation ranges across the event phases, whereas OA and HYP exhibited relatively smaller variations. This suggests that CA and MA events trigger more pronounced rapid, short-term fluctuations in heart rate.

Heart rate (HR) also exhibited consistent dynamic patterns across all respiratory event types. Specifically, HR decelerated during the events and subsequently accelerated upon the cessation of the sleep-disordered breathing (SDB) events, ultimately exceeding pre-event baseline levels (Figure 2—left). Notably, the OA group displayed the greatest amplitude of HR fluctuation (Figure 2—right). Meanwhile, QRS duration demonstrated a distinct pattern, characterized by prolongation during the respiratory event followed by a decline toward baseline after SDB cessation.

### 3.3. HRV Changes Under Severities and Sleep Stages

The linear mixed-effects model (LMM) incorporating respiratory event types, AHI severity, and their interaction terms revealed that AHI severity showed no significant independent association with overall HRV metrics. However, within the severe stratum, the impacts of CA events on HR and QRS duration, as well as the impact of MA events on QRS duration, exhibited significant differences (*p* < 0.05). Post hoc pairwise comparisons further indicated that significant differences among the distinct respiratory event types were exclusively observed within the severe subgroup. For instance, compared to OA and HYP, CA exerted the most potent inhibitory effect on HR. Furthermore, compared to OA, CA was associated with significantly decreased SDSD, RMSSD, and SD1. Additionally, among patients with severe subgroup, MA emerged as the event type inducing the most pronounced intraventricular conduction delay (indicated by prolonged QRS), with its effect surpassing even that of CA (Tables S2–S4).

Furthermore, the LMM incorporating the interaction between respiratory event type and sleep stage revealed that the differences in electrocardiographic features across event types were strongly sleep-stage dependent.

EMM-based pairwise comparisons demonstrated that significant differences between respiratory event types were primarily observed during NREM sleep. Specifically, OA showed significantly higher HRV indices than CA, including SDSD (estimate = 11.69, *p* < 0.001), RMSSD (estimate = 11.59, *p* < 0.001), and SD1 (estimate = 8.26, *p* < 0.001), along with higher HR (estimate = 1.12, *p* < 0.001). In addition, significant differences were also observed between OA and MA in HR (estimate = 0.59, *p* < 0.001), while other comparisons showed weaker or non-significant effects (Table 5 and Tables S5 and S6).

In contrast, during REM sleep, no significant differences in HRV indices, heart rate, or QRS duration were detected between respiratory event types after multiple-comparison correction, indicating that the inter-event heterogeneity observed in NREM sleep was largely attenuated during REM sleep.

## 4. Discussion

This study, for the first time, demonstrates the heterogeneous effects of distinct respiratory event subtypes on cardiac electrophysiological activity and further provides preliminary evidence regarding the modulatory roles of disease severity and sleep stage in these dynamics. Our findings indicate that respiratory event subtype is a key determinant of HRV alterations. Specifically, CA exerted the strongest inhibitory effect on HRV, whereas MA predominantly impaired ventricular conduction, as reflected by QRS prolongation. In contrast, OA and HYP showed largely comparable effects on cardiac electrophysiology. Furthermore, we identified distinct regulatory patterns underlying these effects: the adverse cardiac effects associated with CA were jointly influenced by disease severity and sleep stage, whereas the effects of MA appeared to depend primarily on disease severity and were largely independent of sleep stage.

Compared with OA, CA events were associated with significantly reduced HRV and greater heart rate deceleration, suggesting a stronger inhibitory effect on autonomic regulation. This phenomenon may be attributable to the unique pathophysiological mechanism of CA, which is primarily characterized by suppression of central respiratory drive. Previous studies have shown that during CA events, the high-frequency (HF) component of HRV, which reflects parasympathetic activity, may nearly disappear and subsequently resynchronize with respiratory rhythms after ventilation resumes, without substantial compensatory activation of the low-frequency (LF) component related to sympathetic regulation [22]. In the present study, time-domain HRV indices such as RMSSD and SDSD mainly reflected cardiac vagal (parasympathetic) modulation. CA events appeared to selectively suppress parasympathetic regulation without eliciting a compensatory sympathetic response, ultimately resulting in a greater reduction in HRV magnitude compared with OA and MA events.

Although Szollosi I et al. [31] did not independently evaluate the effects of OA and CA on HRV, they observed in patients with congestive heart failure (CHF) that, compared to OSA, CSA patients exhibited lower SDNN, absolute HF power, and HF percentage during SDB segments (two 10 min segments in NREM sleep). While the present study did not assess absolute HF and LF powers, HF oscillations are mathematically and physiologically parallel to RMSSD. The concurrent decline in HF oscillations and RMSSD indices reflects an impaired capacity for short-term heart rate variability, indicating diminished parasympathetic activity. This finding is consistent with the inhibitory effect of CA on HRV observed in our cohort.

Our findings differ somewhat from those reported by Spicuzza L et al. [22], who observed significantly higher HF oscillations during OA than during post-apnea hyperventilation. In contrast, we did not identify significant event-type-dependent differences in parasympathetic activity, as represented by RMSSD. Instead, all respiratory event subtypes exhibited a similar dynamic HRV pattern, characterized by HRV reduction during SDB events followed by compensatory rebound after event termination. Similarly, Hietakoste S et al. [32] reported increased RMSSD values after apneas and hypopneas compared with intra-event periods; however, their analysis did not distinguish among central, obstructive, and mixed apnea subtypes. Chouchou F et al. [33] further proposed that post-event cardiac sympathetic modulation is primarily driven by cortical arousals and hypoxia rather than by respiratory event subtype or duration. Interestingly, although OA and HYP exhibited comparable event durations and OA was associated with greater oxygen desaturation, their overall effects on HRV were not significantly different. In contrast, CA events, despite shorter durations and milder hypoxic burden, exerted the strongest influence on HRV metrics. These findings suggest that respiratory event subtype may represent a more important determinant of HRV alterations than conventional factors such as event duration, arousal burden, or hypoxemia severity.

Notably, the effects of MA events on cardiac electrophysiology were primarily manifested as intraventricular conduction delay, reflected by significant QRS prolongation. This effect became more pronounced in patients with severe SDB and appeared to be independent of sleep stage. MA events involve a dual pathophysiological burden, namely central respiratory drive suppression combined with increased upper airway resistance. This combined burden may contribute to intraventricular conduction delay through mechanisms such as myocardial electrophysiological remodeling and ion channel dysfunction.

Given that CA and MA account for approximately 30% of all apnea events even among patients with OSA [34], the conventional strategy adopted in previous studies [32,33] of grouping all apnea events into a single category may overlook the substantial heterogeneity among respiratory event subtypes. Different respiratory event types may exert fundamentally distinct effects on heart rate regulation, ventricular conduction, and autonomic balance. Consequently, analyses that aggregate all apnea events may obscure subtype-specific cardiovascular effects and limit a comprehensive understanding of the mechanisms underlying SDB-related cardiac injury.

Additionally, our study revealed that the differences in HRV across distinct respiratory event types predominantly emerged during the NREM sleep stage. During REM sleep, no significant differences were observed across event types regarding HRV metrics, heart rate, or QRS duration (all adjusted *p* > 0.05), indicating a marked attenuation of differential cardiac electrophysiological impacts during this phase. Physiologically, NREM sleep is characterized by enhanced parasympathetic tone and reduced sympathetic activity. As individuals transition from wakefulness to NREM sleep, autonomic function stabilizes, accompanied by reductions in blood pressure, heart rate, and systemic vascular resistance. Conversely, REM sleep is characterized by abrupt surges in sympathetic activity, resulting in profound heart rate fluctuations and irregular respiratory patterns, intrinsically coupled with a withdrawal of parasympathetic tone [6,7,23]. Consequently, most studies report a decline in parasympathetic-related HRV indices (e.g., HF, RMSSD, pNN50) during REM sleep [8,23]. Spicuzza L et al. [22] also corroborated that HF oscillation power during and after apneas—whether CA or OA—was lower in REM sleep compared to NREM sleep. In the present study, the significant interaction between CA events and sleep stage indicates that the influence of CA on HRV is sleep-stage dependent. Stable parasympathetic predominance during NREM sleep establishes a steady physiological baseline, allowing clear detection of distinct HRV differences between CA and OA. In contrast, REM sleep is characterized by inherent autonomic lability and fluctuating sympathetic tone, which obscure the differential HRV responses to CA versus OA and render intergroup comparisons non-significant during the REM period.

Unlike previous studies, we did not find a direct association between baseline HRV indices and overall AHI severity. The impact of disease severity on cardiac electrical activity was primarily manifested through its interaction with respiratory event types. For example, within the severe subgroup, CA events significantly impacted heart rate, while both CA and MA events significantly altered QRS duration. Nevertheless, the statistical robustness of these interaction terms was relatively limited, indicating that, compared to the dominant main effect of respiratory event types, the influence of disease severity and its interaction with event types on HRV remains relatively modest.

This study has certain limitations that warrant consideration. First, since most of the SDB event durations ranged from 20 to 40 s, frequency-domain HRV analysis (such as Low Frequency [LF] and High Frequency [HF] power) could not be performed. According to standard HRV signal processing guidelines, frequency-domain analysis requires longer signal windows (typically at least 1 to 2 min) to achieve the necessary spectral resolution, particularly for capturing LF oscillations (0.04–0.15 Hz). Consequently, changes in sympathetic excitability could not be directly evaluated through spectral bands, and our analysis was necessarily restricted to validated ultra-short-term time-domain and non-linear metrics. Second, the study population was predominantly male, which may introduce potential gender bias and limit the generalizability of the findings. Therefore, future studies with more balanced and larger cohorts are needed to validate whether the observed differences in HRV features across respiratory event types are consistent across sexes. Finally, cortical arousals associated with the termination of respiratory events may also profoundly influence HRV results. Although we meticulously designed our signal windows to focus on the intra-event period, the complex interplay between hypoxia-driven autonomic changes and arousal-induced sympathetic surges remains difficult to fully decouple without high-resolution synchronous EEG analysis. This requires further study in the future.

## 5. Conclusions

In summary, the findings of this study not only fill the research gap in prior studies that treated all apnea events as a homogeneous category but also highlight the importance of accounting for respiratory event heterogeneity when evaluating SDB-related cardiovascular risk. This provides a novel perspective for precise clinical identification of high-risk individuals within the SDB population. Further, the proportion of CA events across different sleep stages may serve as valuable supplementary biomarkers to the traditional AHI metric. Such a framework may help refine risk stratification for SDB-associated cardiovascular complications and potentially provide a reasonable theoretical basis for the future development of individualized, targeted clinical intervention strategies.

## Figures and Tables

**Table 1:** The HRV feature information of the research.

HRV Feature	Description	Unit	Physiological Meaning
SDSD	Standard deviation of successive RR interval differences	ms	Primarily reflects the activity of the parasympathetic nervous system (vagus nerve) and is associated with short-term heart rate variability. A smaller value indicates a decline in short-term heart rate stability.
RMSSD	Root mean square of successive RR differences	ms	Focuses more on short-term heart rate variability, specifically capturing vagally mediated heart rate fluctuations during the respiratory cycle. A decrease in value mainly suggests weakened vagal activity or impaired function. A decline in RMSSD is a marker of poor prognosis, suggesting an increased risk of malignant arrhythmias (such as ventricular fibrillation) and sudden death [28].
SD1	Short axis of Poincaré plot	ms	Reflects short-term fluctuations in heart rate, similar to RMSSD, and is primarily associated with vagal activity. A decrease in value indicates attenuated vagal activity.
SDNN	Standard Deviation of Successive RR intervals	ms	A global HRV index reflecting total heart rate variability. A decrease in value typically suggests impaired cardiac autonomic regulatory function. Reduced SDNN is associated with poor prognosis in various cardiovascular diseases and serves as an independent risk predictor.
SD2	Long axis of Poincaré plot	ms	Reflects long-term fluctuations in heart rate and is associated with the complexity of global autonomic regulation. A decrease in value suggests impaired long-term cardiac regulatory function.
QRS Duration	Duration of QRS complex	ms	Refers to the duration of the QRS complex on the electrocardiogram, reflecting the synchrony of ventricular depolarization. A QRS duration ≥ 130 ms is a strong predictor of pVT [29].

RMSSD and SDSD are commonly used statistical metrics; the former reflects all cyclic components contributing to heart rate variability, primarily representing overall autonomic variability; the latter reflects short-term rapid fluctuations in heart rate, representing the regulatory function of the parasympathetic nervous system (vagus nerve) on the heart and the balance status of the ANS.

**Table 2:** Baseline characteristics of patients.

Variables	5 ≤ AHI < 15 n = 41	15 ≤ AHI < 30 n = 17	AHI ≥ 30 n = 50	Total n = 108	*p*
Age, years	41.2 ± 13.5	47.2 ± 16.0	51.9 ± 13.6	47.1 ± 14.9	0.002
Male: Female	37:4	16:1	46:4	99:9	0.539
BMI, kg/m^2^	26.9 ± 3.7	25.7 ± 3.5	26.7 ± 2.9	36.7 ± 2.9	0.475
Comorbidity, N (%)					
Hypertension	9 (22.0)	5 (29.4)	19 (38.0)	33 (30.1)	0.309
Treated with antihypertensive drugs	9 (22.0)	5 (29.4)	18 (36.0)	32 (29.6)	0.370
Coronary artery disease	1 (2.4)	1 (5.9)	6 (12.0)	8 (7.4)	0.127
Treated with β-receptor blockers	0 (0)	1 (5.9)	4 (8.0)	5 (4.6)	0.058
Treated with BZRAs ^1^	2 (4.9)	1 (5.9)	3 (6.0)	6 (5.6)	0.851
ESS	7.00 (4.00–12.00)	4.50 (3.75–13.75)	7.00 (4.00–12.25)	7.00 (4.00–13.00)	0.602
FS-14	6.68 ± 3.80	7.25 ± 3.68	5.55 ± 3.23	6.25 ± 3.56	0.153
BAI	24.00 (22.00–29.00)	27.00 (23.00–31.00)	24.00 (22.00–28.00)	24.00 (22.00–29.00)	0.191
BDI	8.00 (4.00–16.00)	12.00 (8.50–14.25)	8.50 (5.00–13.00)	9.00 (4.00–14.00)	0.515
Baseline PSG data					
TST, min	413.88 ± 60.37	400.59 ± 75.37	354.29 ± 87.32	384.20 ± 80.63	0.001
SE, %	85.30 (80.50–91.30)	79.80 (75.30–83.10)	74.90 (65.82–87.03)	80.80 (72.10–88.25)	<0.001
SL, min	5.00 (1.50–11.50)	7.50 (3.00–18.50)	8.00 (4.12–19.25)	6.25 (2.38–15.12)	0.187
WASO, min	61.50 (35.50–78.00)	95.50 (56.00–120.00)	106.25 (56.12–161.12)	75.25 (46.75–123.38)	0.001
N1, %	12.4 ± 5.5	15.1 ± 7.8	31.7 ± 14.8	21.8 ± 14.5	<0.001
N2, %	41.7 ± 9.3	46.3 ± 7.0	41.9 ± 10.9	42.5 ± 9.8	0.222
N3, %	18.6 ± 7.4	15.9 ± 7.9	8.3 ± 7.0	13.4 ± 8.7	<0.001
REM, %	27.3 ± 7.7	22.7 ± 5.1	18.2 ± 9.0	22.3 ± 9.0	<0.001
AHI, events/hr	9.4 ± 2.9	19.7 ± 2.3	55.6 ± 17.2	42.0 ± 16.4	<0.001
Average SpO_2_, %	96.00 (95.00–96.00)	95.00 (94.00–95.00)	92.00 (89.25–93.00)	94.00 (92.00–95.00)	<0.001
Min SpO_2_, %	86.00 (84.00–89.00)	86.00 (85.00–89.00)	78.50 (69.25–84.00)	84.00 (77.00–88.00)	<0.001
CT90 ^2^, min	1.28 (0.23–3.57)	1.65 (0.67–5.32)	23.35 (9.53–68.25)	5.22 (1.08–25.01)	<0.001

^1^ Note: BZRA refers to benzodiazepine receptor agonists, a class of hypnotic agents that act on the γ-aminobutyric acid type A (GABA_A) receptor complex. BZRA medications are widely used for the treatment of insomnia due to their sedative, hypnotic, anxiolytic and muscle-relaxant effects. ^2^ Note: CT90 refers to the cumulative time during sleep when the blood oxygen saturation (SpO_2_) falls below 90%. CT90 is widely used as an index of nocturnal hypoxemia and is strongly associated with the severity of obstructive sleep apnea and cardiovascular risk.

**Table 3:** Estimated marginal means (EMMs) derived from linear mixed-effects models and Sidak-adjusted pairwise comparisons of HRV features across respiratory event types.

Pairwise_Comparison	OA–CA	OA–MA	OA–HYP	CA–MA	CA–HYP	MA–HYP
SDSD Estimate [95% CI] ^1^	9.17 [2.88, 15.46]	3.97 [−0.66, 8.60]	0.17 [−4.57, 4.90]	−5.20 [−11.85, 1.45]	−9.00 [−15.38, −2.62]	−3.80 [−9.23, 1.63]
SDSD *p* value	**<0.001**	0.137	1	0.215	**0.001**	0.334
SDNN Estimate [95% CI]	4.99 [−7.41, 17.39]	−0.10 [−9.29, 9.08]	0.79 [−8.50, 10.09]	−5.09 [−18.24, 8.05]	−4.20 [−16.77, 8.38]	0.90 [−9.76, 11.56]
SDNN *p* value	0.872	1	1	0.89	0.943	1
RMSSD Estimate [95% CI]	9.04 [2.41, 15.67]	3.82 [−1.06, 8.71]	0.39 [−4.60, 5.39]	−5.21 [−12.23, 1.80]	−8.64 [−15.38, −1.91]	−3.43 [−9.16, 2.30]
RMSSD *p* value	**0.002**	0.215	1	0.267	**0.004**	0.521
HR Estimate [95% CI]	0.86 [0.49, 1.22]	0.47 [0.20, 0.74]	−0.13 [−0.40, 0.15]	−0.39 [−0.78, −0.00]	−0.98 [−1.36, −0.61]	−0.59 [−0.91, −0.28]
HR *p* value	**<0.001**	**<0.001**	0.792	**0.048**	**<0.001**	**<0.001**
SD1 Estimate [95% CI]	6.48 [2.04, 10.93]	2.81 [−0.47, 6.08]	0.12 [−3.23, 3.47]	−3.68 [−8.38, 1.02]	−6.37 [−10.88, −1.85]	−2.69 [−6.53, 1.15]
SD1 *p* value	**<0.001**	0.137	1	0.215	**<0.001**	0.334
SD2 Estimate [95% CI]	4.51 [−10.70, 19.71]	−1.39 [−12.69, 9.91]	0.30 [−11.06, 11.67]	−5.90 [−22.03, 10.24]	−4.20 [−19.62, 11.21]	1.70 [−11.34, 14.73]
SD2 *p* value	0.968	1	1	0.914	0.979	1
QRS Duration Estimate [95% CI]	−0.24 [−0.93, 0.44]	−0.52 [−1.03, −0.02]	0.23 [−0.29, 0.74]	−0.28 [−1.01, 0.45]	0.47 [−0.23, 1.17]	0.75 [0.15, 1.34]
QRS Duration *p* value	0.926	**0.038**	0.826	0.892	0.384	**0.005**

^1^ Note: Estimate represents the fixed-effect coefficient derived from the linear mixed-effects model; CI, 95% confidence interval; *p* value, *p*-value for statistical significance. Statistical significance was defined as *p* < 0.05.

**Table 4:** Comparison of HRV characteristics before and after sleep apnea events.

Feature	Time Range	OA	CA	MA	HYP	*p* Value
		N = 2830	N = 815	N = 2204	N = 1662	
SDSD, ms	Pre Event	27.42 (17.65–46.94)	25.75 (17.08–45.79)	32.68 (19.21–77.11)	25.25 (15.16–46.53)	<0.001
	Event	22.43 (14.32–35.58)	13.30 (8.79–23.54)	20.85 (11.87–43.00)	22.06 (13.25–36.24)	<0.001
	Post Event	30.66 (18.48–62.59)	31.33 (19.34–56.76)	37.90 (20.72–86.41)	27.14 (16.45–52.93)	<0.001
	*p*-value	<0.001	<0.001	<0.001	<0.001	
RMSSD, ms	Pre Event	28.37 (18.59–48.39)	26.98 (18.34–46.91)	34.48 (20.41–80.03)	25.65 (15.73–47.32)	<0.001
	Event	22.94 (15.02–36.37)	13.96 (9.50–25.28)	21.48 (12.67–44.86)	22.40 (13.57–36.59)	<0.001
	Post Event	31.29 (19.01–63.56)	32.05 (19.59–57.85)	38.59 (21.15–88.32)	27.45 (16.93–53.56)	<0.001
	*p*-value	<0.001	<0.001	<0.001	<0.001	
SD1, ms	Pre Event	19.39 (12.48–33.19)	18.21 (12.08–32.38)	23.11 (13.59–54.53)	17.85 (10.72–32.90)	<0.001
	Event	15.86 (10.12–25.16)	9.40 (6.21–16.64)	14.75 (8.39–30.40)	15.60 (9.37–25.62)	<0.001
	Post Event	21.68 (13.07–44.26)	22.15 (13.67–40.14)	26.80 (14.65–61.10)	19.19 (11.63–37.42)	<0.001
	*p*-value	<0.001	<0.001	<0.001	<0.001	
HR, bpm	Pre Event	67.14 (59.58–75.56)	67.17 (60.46–74.31)	66.35 (57.91–75.42)	63.43 (58.83–72.25)	<0.001
	Event	63.35 (57.01–72.02)	60.45 (56.75–66.99)	62.17 (54.33–69.87)	60.30 (56.23–68.94)	<0.001
	Post Event	71.42 (62.55–79.66)	67.60 (61.92–74.77)	69.34 (61.54–77.89)	64.30 (59.45–73.31)	<0.001
	*p*-value	<0.001	<0.001	<0.001	<0.001	
QRS duration, ms	Pre Event	97.78 (80.08–105.19)	94.14 (82.52–103.40)	91.19 (73.80–103.66)	102.40 (87.17–108.77)	<0.001
	Event	99.48 (82.73–106.72)	98.21 (85.36–106.41)	97.43 (82.31–107.14)	103.38 (87.59–109.79)	<0.001
	Post Event	96.40 (80.31–104.00)	95.61 (83.89–103.66)	91.27 (75.83–102.86)	101.89 (86.43–108.17)	<0.001
	*p*-value	<0.001	<0.001	<0.001	<0.001	

**Table 5:** Estimated marginal means derived from linear mixed-effects models and Sidak-adjusted pairwise comparison of HRV features in each sleep stage (NREM and REM).

Feature	OA–CA	OA–MA	OA–HYP	CA–MA	CA–HYP	MA–HYP
**Stage: NREM**						
SDSD Estimate [95% CI]	11.69 [4.59, 18.78]	4.80 [−0.33, 9.93]	0.31 [−4.97, 5.59]	−6.89 [−14.31, 0.53]	−11.38 [−18.55, −4.20]	−4.49 [−10.40, 1.42]
SDSD *p* value	**<0.001**	0.08	1	0.085	**<0.001**	0.245
SDNN Estimate [95% CI]	7.43 [−6.60, 21.45]	0.19 [−10.00, 10.37]	0.56 [−9.83, 10.95]	−7.24 [−21.94, 7.46]	−6.87 [−21.04, 7.31]	0.37 [−11.26, 12.01]
SDNN *p* value	0.657	1	1	0.728	0.743	1
RMSSD Estimate [95% CI]	11.59 [4.10, 19.08]	4.62 [−0.79, 10.03]	0.52 [−5.05, 6.09]	−6.97 [−14.81, 0.86]	−11.07 [−18.64, −3.50]	−4.10 [−10.34, 2.14]
RMSSD *p* value	**<0.001**	0.139	1	0.11	**<0.001**	0.409
HR Estimate [95% CI]	1.12 [0.71, 1.53]	0.59 [0.29, 0.89]	−0.18 [−0.48, 0.13]	−0.53 [−0.96, −0.10]	−1.30 [−1.72, −0.88]	−0.77 [−1.11, −0.42]
HR *p* value	**<0.001**	**<0.001**	0.564	**0.007**	**<0.001**	**<0.001**
SD1 Estimate [95% CI]	8.26 [3.25, 13.28]	3.39 [−0.23, 7.02]	0.22 [−3.51, 3.95]	−4.87 [−10.12, 0.38]	−8.04 [−13.12, −2.97]	−3.17 [−7.35, 1.01]
SD1 *p* value	**<0.001**	0.08	1	0.085	**<0.001**	0.245
SD2 Estimate [95% CI]	6.89 [−10.33, 24.11]	−1.01 [−13.54, 11.52]	0.09 [−12.63, 12.81]	−7.90 [−25.96, 10.16]	−6.79 [−24.19, 10.61]	1.10 [−13.14, 15.35]
SD2 *p* value	0.875	1	1	0.822	0.887	1
QRS Duration Estimate [95% CI]	0.01 [−0.76, 0.79]	−0.50 [−1.06, 0.06]	0.24 [−0.34, 0.81]	−0.51 [−1.32, 0.30]	0.22 [−0.56, 1.01]	0.74 [0.09, 1.38]
QRS Duration *p* value	1	0.105	0.864	0.455	0.973	**0.016**
**Stage: REM**						
SDSD Estimate [95% CI]	1.46 [−10.34, 13.26]	0.98 [−8.66, 10.63]	−0.05 [−8.33, 8.23]	−0.48 [−13.64, 12.69]	−1.51 [−13.61, 10.58]	−1.04 [−11.33, 9.26]
SDSD *p* value	1	1	1	1	1	1
SDNN Estimate [95% CI]	−2.31 [−25.71, 21.09]	−0.80 [−20.01, 18.42]	2.07 [−14.34, 18.48]	1.52 [−24.63, 27.67]	4.38 [−19.57, 28.33]	2.86 [−17.56, 23.29]
SDNN *p* value	1	1	1	1	0.997	0.999
RMSSD Estimate [95% CI]	1.23 [−11.23, 13.68]	1.00 [−9.18, 11.18]	0.26 [−8.47, 9.00]	−0.23 [−14.12, 13.67]	−0.96 [−13.72, 11.80]	−0.73 [−11.60, 10.13]
RMSSD *p* value	1	1	1	1	1	1
HR Estimate [95% CI]	−0.07 [−0.76, 0.62]	−0.12 [−0.68, 0.44]	−0.18 [−0.66, 0.30]	−0.05 [−0.81, 0.72]	−0.11 [−0.82, 0.59]	−0.07 [−0.66, 0.53]
HR *p* value	1	0.995	0.899	1	0.999	1
SD1 Estimate [95% CI]	1.03 [−7.31, 9.38]	0.70 [−6.13, 7.52]	−0.04 [−5.89, 5.82]	−0.34 [−9.65, 8.97]	−1.07 [−9.62, 7.48]	−0.73 [−8.01, 6.54]
SD1 *p* value	1	1	1	1	1	1
SD2 Estimate [95% CI]	−2.53 [−31.31, 26.25]	−2.35 [−26.03, 21.32]	1.61 [−18.56, 21.78]	0.18 [−32.01, 32.37]	4.14 [−25.31, 33.59]	3.96 [−21.15, 29.08]
SD2 *p* value	1	1	1	1	0.999	0.999
QRS Duration Estimate [95% CI]	−1.07 [−2.36, 0.22]	−0.62 [−1.67, 0.43]	0.11 [−0.79, 1.02]	0.45 [−0.99, 1.88]	1.18 [−0.14, 2.50]	0.73 [−0.39, 1.86]
QRS Duration *p* value	0.163	0.536	1	0.959	0.107	0.414

## Data Availability

The data presented in this study are not publicly available due to privacy and ethical restrictions. Data may be available from the corresponding author upon reasonable request and with permission from the relevant ethics committee.

## Supplementary Materials

The following supporting information can be downloaded at: https://www.mdpi.com/article/10.3390/diagnostics16121770/s1, Table S1: All parameters in the main LMM; Table S2: Linear mixed-effect models in interaction with different subject severity (Mild, Moderate and Severe) for usHRV features; Table S3: Estimated marginal means (EMMs) and Sidak-adjusted pairwise comparisons of usHRV features among severity groups stratified by respiratory event type; Table S4: Estimated marginal means (EMMs) and Sidak-adjusted pairwise comparisons of usHRV features among respiratory event types stratified by severity (Mild, Moderate, and Severe); Table S5: Linear mixed-effect models in interaction with different stages(NREM vs. REM) for usHRV features; Table S6: Estimated marginal means (EMMs) derived from linear mixed-effects models and Sidak-adjusted pairwise comparison of usHRV features between sleep stages (NREM vs. REM) within each respiratory event type.

## Abbreviation

The following abbreviations are used in this manuscript:

OA	Obstructive Apnea
CA	Central Apnea
MA	Mixed Apnea
HYP	Hypopnea
