# Supplementary material for: Differential Effects of Sleep Respiratory Event Types on Heart Rate Variability: Central Apnea as the Most Significant
Source: Diagnostics (Basel). 2026 Jun 8;16(12):1770. doi: 10.3390/diagnostics16121770 (PMC13298131; doi:10.3390/diagnostics16121770)
Supplement: Supplementary file 1 [file diagnostics-16-01770-s001.zip › diagnostics-4324956-supplementary.pdf]

Supplementary Table and Figure

Table S1. All parameters in the main LMM

Factors	SDSD Estimate [95% CI]	SDSD P-value	SDNN Estimate [95% CI]	SDNN P-value	RMSSD Estimate [95% CI]	RMSSD P-value	HR Estimate [95% CI]	HR P-value	SD1 Estimate [95% CI]	SD1 P-value	SD2 Estimate [95% CI]	SD2 P-value	QRS_DURATION Estimate [95% CI]	QRS_DURATION P-value
OA	90.53 [1.84, 179.22]	-	102.11 [23.38, 180.83]	-	92.54 [2.53, 182.55]	-	52.07 [34.69, 69.45]	< 0.001	64.01 [1.30, 126.73]	-	118.26 [36.52, 200.00]	-	51.95 [11.56, 92.33]	-
CA	-9.17 [-13.85, -4.49]	**< 0.001**	-4.99 [-14.22, 4.25]	0.29	-9.04 [-13.98, -4.10]	**< 0.001**	-0.86 [-1.13, -0.58]	**< 0.001**	-6.48 [-9.80, -3.17]	**< 0.001**	-4.51 [-15.84, 6.82]	0.435	0.24 [-0.27, 0.75]	0.353
MA	-3.97 [-7.42, -0.52]	**0.024**	0.10 [-6.74, 6.95]	0.976	-3.82 [-7.46, -0.18]	**0.04**	-0.47 [-0.67, -0.27]	**< 0.001**	-2.81 [-5.25, -0.37]	**0.024**	1.39 [-7.03, 9.81]	0.746	0.52 [0.15, 0.90]	**0.006**
HYP	-0.17 [-3.69, 3.36]	0.926	-0.79 [-7.72, 6.13]	0.822	-0.39 [-4.12, 3.33]	0.835	0.13 [-0.08, 0.33]	0.23	-0.12 [-2.61, 2.38]	0.926	-0.30 [-8.77, 8.16]	0.944	-0.23 [-0.61, 0.16]	0.253
BMI	-1.77 [-4.62, 1.07]	0.22	-1.77 [-4.28, 0.74]	0.164	-1.81 [-4.70, 1.08]	0.217	0.65 [0.09, 1.21]	0.023	-1.25 [-3.26, 0.76]	0.22	-1.89 [-4.49, 0.71]	0.153	1.06 [-0.24, 2.36]	0.108
Age	-0.09 [-0.81, 0.63]	0.811	-0.32 [-0.96, 0.31]	0.312	-0.09 [-0.82, 0.64]	0.808	-0.13 [-0.27, 0.02]	0.08	-0.06 [-0.57, 0.45]	0.811	-0.51 [-1.16, 0.14]	0.124	0.30 [-0.03, 0.62]	0.076
Arousal	0.50 [-2.43, 3.43]	0.737	2.16 [-3.65, 7.96]	0.466	0.55 [-2.54, 3.64]	0.727	-0.50 [-0.67, -0.33]	< 0.001	0.35 [-1.72, 2.43]	0.737	3.34 [-3.79, 10.47]	0.359	-0.15 [-0.47, 0.17]	0.348
Hypertension	-12.43 [-34.47, 9.60]	0.265	-7.68 [-26.75, 11.40]	0.426	-12.62 [-34.97, 9.74]	0.265	2.59 [-1.75, 6.94]	0.239	-8.79 [-24.37, 6.79]	0.265	-4.86 [-24.42, 14.70]	0.623	-3.48 [-13.58, 6.61]	0.495
CAD	79.39 [41.36, 117.41]	**< 0.001**	52.04 [19.58, 84.51]	**0.002**	80.07 [41.51, 118.63]	**< 0.001**	1.04 [-6.48, 8.56]	0.785	56.13 [29.25, 83.02]	**< 0.001**	41.91 [8.81, 75.00]	**0.014**	6.95 [-10.53, 24.44]	0.432

Table S2. Linear mixed-effect models in interaction with different subject severity (Mild, Moderate and Severe) for usHRV features .

Factors	SDSD Estimate [95% CI]	SDSD P-value	SDNN Estimate [95% CI]	SDNN P-value	RMSSD Estimate [95% CI]	RMSSD P-value	HR Estimate [95% CI]	HR P-value	SD1 Estimate [95% CI]	SD1 P-value	SD2 Estimate [95% CI]	SD2 P-value	QRS_DURATION Estimate [95% CI]	QRS_DURATION P-value
OA	79.375 [-10.301, 169.052]	-	88.190 [7.805, 168.575]	-	80.718 [-10.288, 171.724]	0.082	52.349 [34.866, 69.833]	-	56.127 [-7.284, 119.538]	-	104.132 [19.740, 188.524]	-	53.799 [12.528, 95.069]	-
CA	-8.428 [-19.883, 3.027]	0.149	-3.576 [-25.891, 18.738]	0.753	-8.049 [-20.133, 4.035]	0.192	-1.358 [-2.029, -0.687]	**< 0.001**	-5.959 [-14.059, 2.141]	0.149	-3.319 [-30.563, 23.926]	0.811	-1.465 [-2.721, -0.210]	**0.022**
MA	-0.465 [-14.892, 13.961]	0.95	4.926 [-23.550, 33.401]	0.735	0.459 [-14.764, 15.683]	0.953	-1.094 [-1.936, -0.252]	**0.011**	-0.329 [-10.530, 9.872]	0.95	6.508 [-28.454, 41.471]	0.715	-1.971 [-3.547, -0.396]	**0.014**
HYP	2.007 [-7.949, 11.962]	0.693	-0.302 [-19.709, 19.105	0.976	1.795 [-8.707, 12.297]	0.738	-0.307 [-0.890, 0.276]	0.302	1.419 [-5.621, 8.458]	0.693	-0.355 [-24.054, 23.345	0.977	-0.203 [-1.293, 0.888]	0.716

Factors	SDSD Estimate [95% CI]	SDSD P-value	SDNN Estimate [95% CI]	SDNN P-value	RMSSD Estimate [95% CI]	RMSSD P-value	HR Estimate [95% CI]	HR P-value	SD1 Estimate [95% CI]	SD1 P-value	SD2 Estimate [95% CI]	SD2 P-value	QRS_DURATION Estimate [95% CI]	QRS_DURATION P-value
			]								]			
severityModerate	16.700 [-13.428, 46.828]	0.275	19.443 [-13.931, 52.817]	0.253	17.848 [-12.894, 48.591]	0.253	1.479 [-4.022, 6.980]	0.595	11.809 [-9.495, 33.112]	0.275	18.863 [-18.892, 56.619]	0.327	-2.444 [-15.397, 10.508]	0.709
severitySevere	1.639 [-20.645, 23.923]	0.884	2.997 [-20.993, 26.987]	0.806	1.748 [-20.973, 24.469]	0.879	2.961 [-1.146, 7.068]	0.156	1.159 [-14.598, 16.916]	0.884	3.667 [-23.215, 30.549]	0.789	-2.280 [-11.954, 7.394]	0.641
Arousal(Yes)	0.492 [-2.450, 3.435]	0.743	2.312 [-3.522, 8.146]	0.437	0.555 [-2.550, 3.660]	0.726	-0.500 [-0.671, -0.328]	**< 0.001**	0.348 [-1.732, 2.429]	0.743	3.526 [-3.649, 10.700]	0.335	-0.194 [-0.514, 0.127]	0.237
BMI	-1.570 [-4.418, 1.279]	0.277	-1.509 [-4.007, 0.990]	0.234	-1.595 [-4.484, 1.294]	0.276	0.646 [0.088, 1.204]	0.024	-1.110 [-3.124, 0.904]	0.277	-1.624 [-4.222, 0.974]	0.218	1.030 [-0.288, 2.348]	0.124
Age	-0.050 [-0.794, 0.694]	0.894	-0.288 [-0.937, 0.362]	0.381	-0.051 [-0.806, 0.703]	0.893	-0.158 [-0.304, -0.012]	0.034	-0.035 [-0.561, 0.491]	0.894	-0.477 [-1.150, 0.197]	0.163	0.311 [-0.034, 0.655]	0.076
Hypertension(Yes)	-13.023 [-35.028, 8.983]	0.243	-8.531 [-27.439, 10.378]	0.372	-13.238 [-35.550, 9.074]	0.242	2.464 [-1.869, 6.798]	0.262	-9.209 [-24.769, 6.352]	0.243	-5.797 [-25.262, 13.669]	0.555	-3.420 [-13.653, 6.812]	0.509
CAD (Yes)	78.787 [40.852, 116.722]	**< 0.001**	51.648 [19.526, 83.770]	**0.002**	79.482 [41.030, 117.933]	**< 0.001**	0.748 [-6.750, 8.245]	0.843	55.711 [28.887, 82.535]	**< 0.001**	41.729 [8.869, 74.589]	**0.013**	7.059 [-10.648, 24.766]	0.431
CA:severityModerate	2.473 [-16.036, 20.982]	0.793	1.951 [-34.546, 38.447]	0.917	1.860 [-17.671, 21.390]	0.852	-0.744 [-1.825, 0.336]	0.177	1.749 [-11.339, 14.836]	0.793	3.534 [-41.251, 48.320]	0.877	0.811 [-1.211, 2.833]	0.432
MA:severityModerate	9.704 [-12.172, 31.580]	0.385	9.171 [-34.138, 52.479]	0.678	8.862 [-14.224, 31.948]	0.452	1.095 [-0.181, 2.371]	0.093	6.862 [-8.607, 22.330]	0.385	5.521 [-47.711, 58.753]	0.839	1.057 [-1.330, 3.444]	0.386
HYP:severityModerate	2.568 [-12.139, 17.275]	0.732	6.566 [-22.316, 35.449]	0.656	2.346 [-13.171, 17.864]	0.767	0.120 [-0.740, 0.979]	0.785	1.816 [-8.583, 12.215]	0.732	8.354 [-27.015, 43.723]	0.643	-0.823 [-2.431, 0.785]	0.316
CA:severitySevere	-0.399 [-13.199, 12.401]	0.951	-1.256 [-26.287, 23.775]	0.922	-0.645 [-14.150, 12.859]	0.925	0.816 [0.067, 1.565]	**0.033**	-0.282 [-9.333, 8.769]	0.951	-1.153 [-31.762, 29.455]	0.941	2.324 [0.922, 3.725]	**0.001**
MA:severitySevere	-4.332 [-19.211, 10.547]	0.568	-5.728 [-35.108, 23.652]	0.702	-5.158 [-20.859, 10.543]	0.52	0.652 [-0.216, 1.521]	0.141	-3.063 [-13.584, 7.458]	0.568	-5.874 [-41.954, 30.205]	0.75	2.745 [1.120, 4.370]	**< 0.001**
HYP:severitySevere	-3.023 [-13.821, 7.775]	0.583	-1.738 [-22.824, 19.347]	0.872	-2.979 [-14.371, 8.412]	0.608	0.507 [-0.125, 1.139]	0.116	-2.138 [-9.773, 5.498]	0.583	-1.580 [-27.343, 24.184]	0.904	-0.203 [-1.386, 0.979]	0.736

Table S3. Estimated marginal means (EMMs) and Sidak-adjusted pairwise comparisons of usHRV features among severity groups stratified by respiratory event type.

Condition_and_Contrast	SDSD Estimate [95% CI]	SDSD P-value	SDNN Estimate [95% CI]	SDNN P-value	RMSSD Estimate [95% CI]	RMSSD P-value	HR Estimate [95% CI]	HR P-value	SD1 Estimate [95% CI]	SD1 P-value	SD2 Estimate [95% CI]	SD2 P-value	QRS_DURATION Estimate [95% CI]	QRS_DURATION P-value
Event [OA]: Mild - Moderate	-16.700 [-53.070, 19.670]	0.616	-19.443 [-59.956, 21.070]	0.581	-17.848 [-54.969, 19.273]	0.58	-1.479 [-8.098, 5.140]	0.933	-11.809 [-37.526, 13.909]	0.616	-18.863 [-64.738, 27.011]	0.694	2.444 [-13.138, 18.027]	0.975
Event [OA]: Mild - Severe	-1.639 [-28.529, 25.250]	0.998	-2.997 [-32.098, 26.103]	0.993	-1.748 [-29.170, 25.675]	0.998	-2.961 [-7.903, 1.981]	0.391	-1.159 [-20.173, 17.855]	0.998	-3.667 [-36.306, 28.972]	0.991	2.280 [-9.358, 13.918]	0.953
Event [OA]: Moderate - Severe	15.061 [-19.573, 49.695]	0.656	16.446 [-18.852, 51.744]	0.604	16.100 [-19.152, 51.353]	0.62	-1.482 [-8.009, 5.044]	0.93	10.650 [-13.840, 35.140]	0.656	15.196 [-23.852, 54.245]	0.729	-0.164 [-15.550, 15.222]	1
Event [CA]: Mild - Moderate	-19.173 [-55.818, 17.471]	0.51	-21.394 [-63.090, 20.302]	0.526	-19.708 [-57.129, 17.714]	0.504	-0.734 [-7.359, 5.890]	0.991	-13.558 [-39.469, 12.354]	0.51	-22.398 [-69.982, 25.186]	0.597	1.633 [-13.956, 17.223]	0.992
Event [CA]: Mild - Severe	-1.240 [-28.161, 25.680]	0.999	-1.741 [-31.177, 27.695]	0.999	-1.102 [-28.561, 26.356]	1	-3.777 [-8.719, 1.165]	0.19	-0.877 [-19.913, 18.159]	0.999	-2.513 [-35.724, 30.697]	0.997	-0.043 [-11.682, 11.595]	1
Event [CA]: Moderate - Severe	17.933 [-17.901, 53.767]	0.547	19.653 [-20.170, 59.476]	0.559	18.605 [-17.958, 55.169]	0.533	-3.043 [-9.591, 3.506]	0.606	12.680 [-12.658, 38.019]	0.547	19.884 [-25.332, 65.100]	0.648	-1.677 [-17.096, 13.742]	0.991
Event [MA]: Mild - Moderate	-26.404 [-65.773, 12.965]	0.293	-28.614 [-79.196, 21.968]	0.442	-26.710 [-67.096, 13.676]	0.305	-2.574 [-9.251, 4.103]	0.735	-18.671 [-46.509, 9.168]	0.293	-24.384 [-83.621, 34.853]	0.693	1.388 [-14.281, 17.056]	0.995
Event [MA]: Mild - Severe	2.693 [-25.925, 31.310]	0.994	2.731 [-32.513, 37.975]	0.997	3.410 [-25.897, 32.718]	0.99	-3.613 [-8.587, 1.361]	0.229	1.904 [-18.332, 22.140]	0.994	2.207 [-38.727, 43.142]	0.999	-0.465 [-12.151, 11.221]	1
Event [MA]: Moderate - Severe	29.097 [-7.507, 65.700]	0.163	31.345 [-11.126, 73.816]	0.216	30.120 [-7.283, 67.524]	0.155	-1.039 [-7.602, 5.524]	0.974	20.575 [-5.308, 46.457]	0.163	26.592 [-22.124, 75.307]	0.473	-1.853 [-17.293, 13.588]	0.989
Event [HYP]: Mild - Moderate	-19.268 [-53.259, 14.722]	0.44	-26.010 [-57.564, 5.545]	0.14	-20.194 [-54.713, 14.325]	0.412	-1.598 [-8.174, 4.978]	0.916	-13.625 [-37.660, 10.410]	0.44	-27.217 [-60.929, 6.494]	0.153	3.267 [-12.251, 18.785]	0.943
Event [HYP]: Mild - Severe	1.384 [-24.298, 27.065]	0.999	-1.259 [-25.916, 23.399]	0.999	1.232 [-24.871, 27.334]	0.999	-3.468 [-8.388, 1.452]	0.252	0.978 [-17.181, 19.138]	0.999	-2.087 [-28.748, 24.573]	0.997	2.484 [-9.122, 14.089]	0.94
Event [HYP]: Moderate - Severe	20.652 [-12.675, 53.979]	0.362	24.751 [-5.204, 54.706]	0.139	21.426 [-12.394, 55.246]	0.342	-1.870 [-8.373, 4.634]	0.869	14.603 [-8.963, 38.169]	0.362	25.130 [-6.461, 56.721]	0.163	-0.784 [-16.136, 14.568]	0.999

Table S4. Estimated marginal means (EMMs) and Sidak-adjusted pairwise comparisons of usHRV features among respiratory event types stratified by severity (Mild, Moderate, and Severe).

Condition_and_Contrast	SDSD Estimate [95% CI]	SDSD P-value	SDNN Estimate [95% CI]	SDNN P-value	RMSSD Estimate [95% CI]	RMSSD P-value	HR Estimate [95% CI]	HR P-value	SD1 Estimate [95% CI]	SD1 P-value	SD2 Estimate [95% CI]	SD2 P-value	QRS_DURATION Estimate [95% CI]	QRS_DURATION P-value
Severity [Mild]: OA - CA	8.428 [-6.948, 23.804]	0.621	3.576 [-26.375, 33.528]	1	8.049 [-8.172, 24.269]	0.721	1.358 [0.457, 2.259]	< 0.001	5.959 [-4.913, 16.832]	0.621	3.319 [-33.249, 39.887]	1	1.465 [-0.220, 3.150]	0.126
Severity [Mild]: OA - MA	0.465 [-18.899, 19.830]	1	-4.926 [-43.147, 33.296]	1	-0.459 [-20.893, 19.975]	1	1.094 [-0.037, 2.224]	0.064	0.329 [-13.364, 14.022]	1	-6.508 [-53.438, 40.421]	0.999	1.971 [-0.143, 4.086]	0.082
Severity [Mild]: OA - HYP	-2.007 [-15.369, 11.356]	0.999	0.302 [-25.747, 26.351]	1	-1.795 [-15.892, 12.302]	1	0.307 [-0.475, 1.090]	0.884	-1.419 [-10.868, 8.030]	0.999	0.355 [-31.456, 32.165]	1	0.203 [-1.262, 1.667]	0.999
Severity [Mild]: CA - MA	-7.962 [-26.389, 10.465]	0.83	-8.502 [-44.972, 27.967]	0.99	-8.508 [-27.954, 10.938]	0.822	-0.264 [-1.339, 0.811]	0.988	-5.630 [-18.660, 7.400]	0.83	-9.827 [-54.666, 35.012]	0.993	0.506 [-1.505, 2.518]	0.986
Severity [Mild]: CA - HYP	-10.434 [-22.771, 1.903]	0.147	-3.275 [-27.296, 20.747]	1	-9.844 [-22.858, 3.171]	0.249	-1.051 [-1.773, -0.328]	< 0.001	-7.378 [-16.102, 1.346]	0.147	-2.964 [-32.289, 26.361]	1	-1.263 [-2.615, 0.089]	0.081
Severity [Mild]: MA - HYP	-2.472 [-19.554, 14.610]	0.999	5.227 [-28.568, 39.023]	0.999	-1.336 [-19.362, 16.690]	1	-0.787 [-1.784, 0.210]	0.207	-1.748 [-13.827, 10.331]	0.999	6.863 [-34.684, 48.410]	0.999	-1.769 [-3.634, 0.096]	0.073
Severity [Moderate]: OA - CA	5.955 [-13.565, 25.474]	0.963	1.626 [-37.158, 40.409]	1	6.189 [-14.412, 26.790]	0.965	2.102 [0.965, 3.240]	< 0.001	4.211 [-9.592, 18.013]	0.963	-0.216 [-47.955, 47.524]	1	0.654 [-1.473, 2.782]	0.961
Severity [Moderate]: OA - MA	-9.239 [-31.315, 12.838]	0.85	-14.096 [-57.918, 29.726]	0.952	-9.321 [-32.620, 13.978]	0.875	-0.001 [-1.288, 1.286]	1	-6.533 [-22.143, 9.078]	0.85	-12.029 [-65.944, 41.885]	0.992	0.915 [-1.492, 3.322]	0.899
Severity [Moderate]: OA - HYP	-4.575 [-19.109, 9.960]	0.957	-6.265 [-35.004, 22.474]	0.993	-4.141 [-19.480, 11.197]	0.98	0.188 [-0.660, 1.035]	0.993	-3.235 [-13.512, 7.043]	0.957	-7.999 [-43.285, 27.286]	0.992	1.026 [-0.561, 2.612]	0.428
Severity [Moderate]: CA - MA	-15.193 [-38.323, 7.937]	0.409	-15.722 [-61.797, 30.353]	0.937	-15.510 [-39.923, 8.902]	0.449	-2.104 [-3.451, -0.756]	< 0.001	-10.743 [-27.099, 5.612]	0.409	-11.814 [-68.605, 44.978]	0.995	0.260 [-2.260, 2.781]	1

Condition_and_Contrast	SDSD Estimate [95% CI]	SDSD P-value	SDNN Estimate [95% CI]	SDNN P-value	RMSSD Estimate [95% CI]	RMSSD P-value	HR Estimate [95% CI]	HR P-value	SD1 Estimate [95% CI]	SD1 P-value	SD2 Estimate [95% CI]	SD2 P-value	QRS_DURATION Estimate [95% CI]	QRS_DURATION P-value
Severity [Moderate]: CA - HYP	-10.529 [-26.936, 5.878]	0.437	-7.890 [-40.513, 24.733]	0.988	-10.330 [-27.646, 6.986]	0.524	-1.915 [-2.871, -0.959]	< 0.001	-7.445 [-19.047, 4.156]	0.437	-7.784 [-47.957, 32.389]	0.996	0.371 [-1.417, 2.160]	0.995
Severity [Moderate]: MA - HYP	4.664 [-14.817, 24.145]	0.989	7.832 [-30.811, 46.475]	0.996	5.180 [-15.379, 25.739]	0.986	0.189 [-0.947, 1.324]	0.999	3.298 [-10.477, 17.073]	0.989	4.030 [-43.500, 51.560]	1	0.111 [-2.014, 2.235]	1
Severity [Severe]: OA - CA	8.827 [1.139, 16.514]	**0.015**	4.833 [-10.439, 20.104]	0.956	8.694 [0.581, 16.807]	**0.029**	0.542 [0.094, 0.990]	0.009	6.241 [0.806, 11.677]	**0.015**	4.472 [-14.319, 23.263]	0.989	-0.858 [-1.696, -0.020]	**0.042**
Severity [Severe]: OA - MA	4.797 [-0.090, 9.685]	0.057	0.803 [-8.899, 10.504]	1	4.699 [-0.460, 9.857]	0.095	0.442 [0.157, 0.727]	< 0.001	3.392 [-0.064, 6.848]	0.057	-0.634 [-12.565, 11.298]	1	-0.774 [-1.307, -0.241]	**< 0.001**
Severity [Severe]: OA - HYP	1.016 [-4.587, 6.620]	0.998	2.040 [-9.012, 13.093]	0.997	1.184 [-4.729, 7.097]	0.996	-0.200 [-0.527, 0.127]	0.496	0.719 [-3.244, 4.681]	0.998	1.934 [-11.611, 15.480]	0.999	0.406 [-0.206, 1.018]	0.397
Severity [Severe]: CA - MA	-4.029 [-11.978, 3.920]	0.701	-4.030 [-19.836, 11.776]	0.985	-3.995 [-12.385, 4.394]	0.757	-0.100 [-0.563, 0.363]	0.994	-2.849 [-8.470, 2.772]	0.701	-5.106 [-24.565, 14.352]	0.982	0.084 [-0.782, 0.951]	1
Severity [Severe]: CA - HYP	-7.810 [-16.217, 0.597]	0.084	-2.792 [-19.438, 13.853]	0.998	-7.510 [-16.382, 1.362]	0.146	-0.742 [-1.232, -0.251]	**< 0.001**	-5.523 [-11.467, 0.422]	0.084	-2.538 [-22.982, 17.906]	1	1.264 [0.347, 2.181]	**0.002**
Severity [Severe]: MA - HYP	-3.781 [-10.076, 2.514]	0.516	1.238 [-11.151, 13.626]	1	-3.514 [-10.157, 3.128]	0.658	-0.642 [-1.009, -0.274]	**< 0.001**	-2.674 [-7.125, 1.778]	0.516	2.568 [-12.596, 17.733]	0.998	1.180 [0.492, 1.867]	**< 0.001**

Table S5. Linear mixed-effect models in interaction with different stages(NREM vs. REM) for usHRV features.

Factors	SDSD Estimate [95% CI]	SDSD P-value	SDNN Estimate [95% CI]	SDNN P-value	RMSSD Estimate [95% CI]	RMSSD P-value	HR Estimate [95% CI]	HR P-value	SD1 Estimate [95% CI]	SD1 P-value	SD2 Estimate [95% CI]	SD2 P-value	QRS_DURATION Estimate [95% CI]	QRS_DURATION P-value
OA	90.60 [1.92, 179.28]	-	102.64 [23.77, 181.51]	-	92.65 [2.64, 182.66]	-	51.69 [34.26, 69.13]	-	64.06 [1.36, 126.77]	-	118.99 [37.04, 200.94]	-	51.80 [11.39, 92.21]	-
event_typeCA	-11.69 [-16.97, -6.40]	< 0.001	-7.43 [-17.88, 3.02]	0.163	-11.59 [-17.17, -6.01]	< 0.001	-1.12 [-1.43, -0.81]	< 0.001	-8.26 [-12.00, -4.53]	< 0.001	-6.89 [-19.72, 5.94]	0.293	-0.01 [-0.59, 0.57]	0.969
event_typeMA	-4.80 [-8.62, -0.98]	0.014	-0.19 [-7.77, 7.40]	0.961	-4.62 [-8.65, -0.59]	0.025	-0.59 [-0.81, -0.37]	< 0.001	-3.39 [-6.09, -0.69]	0.014	1.01 [-8.32, 10.34]	0.832	0.50 [0.08, 0.92]	0.018
event_typeHYP	-0.31 [-4.24, 3.62]	0.876	-0.56 [-8.30, 7.18]	0.887	-0.52 [-4.67, 3.63]	0.806	0.18 [-0.05, 0.41]	0.129	-0.22 [-3.00, 2.56]	0.876	-0.09 [-9.57, 9.39]	0.985	-0.24 [-0.67, 0.19]	0.283
sleep_stageREM	-2.11 [-6.72, 2.50]	0.37	-2.57 [-11.74, 6.61]	0.584	-2.17 [-7.04, 2.69]	0.381	0.55 [0.28, 0.81]	< 0.001	-1.49 [-4.75, 1.77]	0.37	-3.03 [-14.33, 8.27]	0.599	0.15 [-0.35, 0.66]	0.548
Arousal	0.56 [-2.37, 3.49]	0.707	2.17 [-3.64, 7.98]	0.464	0.61 [-2.49, 3.70]	0.7	-0.47 [-0.64, -0.30]	< 0.001	0.40 [-1.68, 2.47]	0.707	3.33 [-3.80, 10.47]	0.36	-0.14 [-0.46, 0.18]	0.403
BMI	-1.76 [-4.61, 1.08]	0.222	-1.76 [-4.27, 0.75]	0.168	-1.80 [-4.69, 1.09]	0.219	0.65 [0.09, 1.21]	0.023	-1.25 [-3.26, 0.77]	0.222	-1.87 [-4.48, 0.73]	0.157	1.06 [-0.24, 2.36]	0.109
Age	-0.08 [-0.80, 0.64]	0.821	-0.33 [-0.96, 0.31]	0.309	-0.09 [-0.82, 0.64]	0.816	-0.12 [-0.26, 0.02]	0.09	-0.06 [-0.57, 0.45]	0.821	-0.52 [-1.17, 0.14]	0.122	0.30 [-0.03, 0.63]	0.074
Hypertension	-12.59 [-34.62, 9.44]	0.259	-7.83 [-26.93, 11.26]	0.417	-12.78 [-35.12, 9.57]	0.259	2.57 [-1.78, 6.93]	0.244	-8.90 [-24.48, 6.68]	0.259	-5.01 [-24.60, 14.59]	0.613	-3.50 [-13.60, 6.60]	0.493
CAD	79.44 [41.43, 117.46]	< 0.001	51.93 [19.42, 84.43]	0.002	80.11 [41.56, 118.66]	< 0.001	1.11 [-6.43, 8.66]	0.77	56.18 [29.30, 83.06]	< 0.001	41.77 [8.61, 74.92]	0.014	6.99 [-10.51, 24.48]	0.43
event_typeCA:sleep_stageREM	10.23 [0.34, 20.12]	0.043	9.74 [-9.92, 29.40]	0.332	10.36 [-0.07, 20.80]	0.052	1.19 [0.62, 1.77]	< 0.001	7.23 [0.24, 14.22]	0.043	9.42 [-14.79, 33.63]	0.446	1.08 [0.00, 2.16]	0.05
event_typeMA:sleep_stageREM	3.82 [-4.12, 11.75]	0.346	0.98 [-14.83, 16.80]	0.903	3.62 [-4.75, 11.99]	0.397	0.71 [0.25, 1.17]	0.003	2.70 [-2.91, 8.31]	0.346	1.34 [-18.16, 20.84]	0.893	0.12 [-0.74, 0.99]	0.785
event_typeHYP:sleep_stageREM	0.37 [-6.41, 7.15]	0.916	-1.51 [-15.01, 12.00]	0.827	0.25 [-6.90, 7.41]	0.944	0.01 [-0.39, 0.40]	0.976	0.26 [-4.54, 5.05]	0.916	-1.52 [-18.16, 15.13]	0.858	0.12 [-0.62, 0.86]	0.746

Table S6. Estimated marginal means (EMMs) derived from linear mixed-effects models and Sidak-adjusted pairwise comparison of usHRV features between sleep stages (NREM vs. REM) within each respiratory event type.

Condition_and_Contrast	SDSD Estimate [95% CI]	SDSD P-value	SDNN Estimate [95% CI]	SDNN P-value	RMSSD Estimate [95% CI]	RMSSD P-value	HR Estimate [95% CI]	HR P-value	SD1 Estimate [95% CI]	SD1 P-value	SD2 Estimate [95% CI]	SD2 P-value	QRS_DURATION Estimate [95% CI]	QRS_DURATION P-value
Event [OA]: NREM - REM	2.11 [-2.50, 6.72]	0.37	2.57 [-6.61, 11.74]	0.584	2.17 [-2.69, 7.04]	0.381	-0.55 [-0.81, -0.28]	< 0.001	1.49 [-1.77, 4.75]	0.37	3.03 [-8.27, 14.33]	0.599	-0.15 [-0.66, 0.35]	0.548
Event [CA]: NREM - REM	-8.12 [-16.94, 0.70]	0.071	-7.17 [-24.68, 10.33]	0.422	-8.19 [-17.50, 1.11]	0.084	-1.74 [-2.25, -1.22]	< 0.001	-5.74 [-11.98, 0.49]	0.071	-6.39 [-27.94, 15.16]	0.561	-1.23 [-2.20, -0.27]	0.012
Event [MA]: NREM - REM	-1.71 [-8.27, 4.86]	0.61	1.58 [-11.49, 14.66]	0.813	-1.45 [-8.37, 5.48]	0.682	-1.25 [-1.63, -0.87]	< 0.001	-1.21 [-5.85, 3.43]	0.61	1.69 [-14.43, 17.80]	0.838	-0.27 [-0.99, 0.44]	0.452
Event [HYP]: NREM - REM	1.74 [-3.31, 6.79]	0.499	4.07 [-5.97, 14.12]	0.427	1.92 [-3.41, 7.25]	0.481	-0.55 [-0.84, -0.26]	< 0.001	1.23 [-2.34, 4.80]	0.499	4.55 [-7.83, 16.92]	0.471	-0.28 [-0.83, 0.27]	0.326
